# An investigation of cerebral oxygen utilization, blood flow and cognition in healthy aging

**DOI:** 10.1371/journal.pone.0197055

**Published:** 2018-05-22

**Authors:** Sarah J. Catchlove, Helen Macpherson, Matthew E. Hughes, Yufen Chen, Todd B. Parrish, Andrew Pipingas

**Affiliations:** 1 Centre for Human Psychopharmacology, Swinburne University, Hawthorn Victoria, Australia; 2 Institute for Physical Activity and Nutrition, Deakin University, Geelong, Victoria, Australia; 3 Centre for Mental Health, Swinburne University, Hawthorn, Victoria, Australia; 4 Australian National Imaging Facility, University of Queensland, St Lucia Queensland, Australia; 5 Feinberg School of Medicine, Northwestern University, Chicago, Illinois, United States of America; Nathan S Kline Institute, UNITED STATES

## Abstract

**Background:**

Understanding how vascular and metabolic factors impact on cognitive function is essential to develop efficient therapies to prevent and treat cognitive losses in older age. Cerebral metabolic rate of oxygen (CMRO_2_), cerebral blood flow (CBF) and venous oxygenation (Y_v_) comprise key physiologic processes that maintain optimum functioning of neural activity. Changes to these parameters across the lifespan may precede neurodegeneration and contribute to age-related cognitive decline. This study examined differences in blood flow and metabolism between 31 healthy younger (<50 years) and 29 healthy older (>50 years) adults; and investigated whether these parameters contribute to cognitive performance.

**Method:**

Participants underwent a cognitive assessment and MRI scan. Grey matter CMRO_2_ was calculated from measures of CBF (phase contrast MRI), arterial and venous oxygenation (TRUST MRI) to assess group differences in physiological function and the contribution of these parameters to cognition.

**Results:**

Performance on memory (p<0.001) and attention tasks (p<0.001) and total CBF were reduced (*p*<0.05), and Y_v_ trended toward a decrease (p = .06) in the older group, while grey matter CBF and CMRO_2_ did not differ between the age groups. Attention was negatively associated with CBF when adjusted (*p*<0.05) in the older adults, but not in the younger group. There was no such relationship with memory. Neither cognitive measure was associated with oxygen metabolism or venous oxygenation in either age group.

**Conclusion:**

Findings indicated an age-related imbalance between oxygen delivery, consumption and demand, evidenced by a decreased supply of oxygen with unchanged metabolism resulting in increased oxygen extraction. CBF predicted attention when the age-effect was controlled, suggesting a task- specific CBF- cognition relationship.

## Introduction

Older age is accompanied by deteriorating cognitive abilities that occurs even in the absence of neurological or neurodegenerative disease [1]. Neuropsychological studies have reported a significant decline in the speed of information processing [2], attention [3, 4], working memory and executive functions [5, 6]. Vascular dysfunction and cardiovascular disease (CVD) also become increasingly prevalent and severe with age, and have been linked to both dementia and cognitive impairment [7–10]. Current evidence suggests that CVD and CVD risk factors such as diabetes, smoking and hypertension can accelerate cognitive decline by causing hypoxia and cerebral hypoperfusion, amongst other pathophysiological processes [11–15]. Likewise, age-related cognitive decline has been attributed to compromised oxygen and nutrient delivery from the cerebral circulation [16, 17], which is highlighted by the observation that inspiration of oxygen-rich gas can significantly improve cognitive performance [18, 19].

In humans, the brain consumes roughly one-fifth of oxygen intake, while only making up about 2% of body weight. Cerebral metabolic rate of oxygen (CMRO_2_), cerebral blood flow (CBF) and venous oxygenation (Y_v_) are measurable indices that reflect brain energy homeostasis. These parameters represent oxygen demand, supply and the portion of oxygen that remains after demand has been met, respectively. Studies of age-related changes in supply and metabolism of oxygen have produced inconsistent results. CMRO_2_ reflects the amount of oxygen used by the brain tissue over time. A number of studies have reported that CMRO_2_ is diminished in older adults [16, 20–28], while other work suggests there is little or no change over the lifespan [29–31]. More recent studies examining aging-related changes in CMRO_2_ reported that the rate actually increased, perhaps due to decreased tissue volume of the elderly brain [32], indeed, remaining neurons would need to expend more energy to maintain the same level of function due to a greater workload [33].

Other research suggests that CMRO_2_ alterations across the lifespan may differ between genders, with males showing an age-related increase while females showed no significant change [32]. Similarly, there are conflicting reports of age-associated changes to the supply of blood to the brain. CBF has been found to decrease with age [24, 25, 34–39], yet others report no differences between younger and older individuals [23, 30, 40–42]. Gender differences [43], brain region [21], and tissue atrophy with age [44], can confound findings of aging effects on CBF. One study reported that cerebral venous oxygenation declined by 1.4% per decade from young adulthood [33], indicating that the oxygen supply/demand equilibrium undergoes a gradual change across the lifespan. It has been demonstrated that age-related changes in CBF and oxygen metabolism are related to performance in healthy adults in calibrated fMRI studies [28, 45]. The present work aims to build upon limited healthy aging studies by examining separate cognitive abilities of memory and attention to investigate whether CBF and CMRO_2_ contribute to cognitive performance in a task-specific manner.

Research indicates that decreased perfusion of the brain may precede and contribute to the onset of clinical dementia [46]. The direct impact of changes in oxygen delivery, consumption and metabolism on performance of memory and attention tasks in cognitively healthy individuals remains to be clarified [47, 48]. Therefore, it is important to establish the magnitude and direction of alterations in vascular and metabolic factors that occur over the healthy lifespan, and the contribution of these parameters to cognition. The present study aimed to investigate the relationships between CBF, CMRO_2_ and cognitive performance in a cognitively intact population. To this end, we used a combination of magnetic resonance imaging and tests of neuropsychological functions to examine differences in CMRO_2_, CBF and cognitive performance between younger (<50 years) and older (>50 years) healthy adults. Based on previous studies [24, 33, 48, 49], it was expected that age-related reductions in CMRO_2_, CBF, as well as cognitive function would be evident. Moreover, we investigated whether CMRO_2_ and CBF were related to cognitive performance on tests of memory and attention.

## Results

Table 1 shows the characteristics of the sample. The two age groups did not differ significantly on measures of blood pressure, arterial oxygen saturation or heart rate. One-way ANOVAs revealed that the groups differed on BMI, tCBF, intracranial and grey matter volumes, and response times on both of the cognitive composite measures, while the differences in venous oxygenation approached significance (*p* = 0.06). Older adults had higher average BMI and response times on the cognitive tasks (i.e. slower processing), yet lower average tCBF, intracranial and grey matter volumes than younger adults.

**Table 1:** Means, standard deviations for characteristics of the entire sample, and each age group separately.

	Whole sample (n = 60) Age range 21–75 years (M 46.9 SD 17.5)	Younger (n = 31)Ages 21–44 years (M29.97 SD6.17)	Older (n = 29) Ages 55–75 years(M65.00 SD5.56)
Years of education	17.2 (3.7)	18.37 (3.22)	15.81 (3.77)
TICS-m			29.35 (2.40)
BMI	24.0 (4.0)**	22.61 (3.21)	25.45 (4.34)
Heart rate	67.0 (9.9)	67.46 (9.63)	66.50 (10.26)
Systolic pressure, mmHg	127.1 (16.3)	124.11 (16.32)	129.90 (16.09)
Diastolic pressure, mmHg	75.8 (10.8)	73.56 (10.69)	77.93 (10.57)
SpO2, %	98.3 (0.8)	98.45 (0.64)	98.21 (.92)
tCBF, ml/min	786.7 (157.1)*	827.33 (147.33)	743.18 (157.99)
gmCBF, ml/100g/min	50.6 (10.2)	51.17 (10.12)	50.02 (10.36)
wmCBF, ml/100g/min	20.25 (4.07)	20.47 (4.05)	20.01 (4.14)
gmCMRO2 μmol/100g/min	148.5 (28.2)	145.98 (30.94)	151.29 (25.18)
Y_v_, %	62.7 (5.4)†	63.94 (5.72)	61.38 (4.77)
ICV, cm^3^	1184.4 (126.1)***	1237.04 (127.34)	1128.09 (98.96)
Vgm, cm^3^	703.8 (84.7)***	751.46 (73.36)	652.83 (64.40)
Vwm, cm^3^	480.9 (56.3)	485.58 (60.44)	475.83 (52.18)
Memory mean RT, msec	908.2 (113.2)***	839.05 (87.08)	982.10 (88.92)
Attention mean RT, msec	529.7 (84.3)***	472.78 (62.32)	586.51 (62.31)

*Note*: Values displayed are M (SD). BMI, body mass index; tCBF, total cerebral blood flow; gmCBF, grey matter cerebral blood flow; CMRO_2_, grey matter cerebral metabolic rate of oxygen; Y_v_, venous oxygenation; ICV, intracranial volume; Vgm, volume of grey matter; Vwm, volume of white matter; TICS-m, modified Telephone Interview for Cognitive Status. Asterisks indicate statistically significant differences between the groups as revealed by one-way ANOVAs.

†*p* = 0.06

*p<0.05

**p<0.01

***p<0.001

### Age and gender effects on physiological measures

Univariate General Linear Models (GLMs) were performed on the data to assess the influences of age and gender on physiological parameters for each age group separately.

In the younger group the main effect of gender approached significance, while age had no association with tCBF. In older adults, gender was again approaching significance, whilst the main effect of age was marginally significant (p = .056). Parameter estimates for the older age group revealed that for each additional year of life, tCBF decreased by approximately 9.70ml/min. The model for the younger group had an R^2^ of .12, while the model for the older group had an R^2^ of .29. Results are shown in Table 2.

**Table 2:** Univariate GLM for each age group, showing the age and gender main effects on tCBF.

Age group		F	p	Partial Eta Squared
Younger	Age	.07	.796	.002
	Gender	3.74	.063	.118
Older	Age	3.99	**.056**	.133
	Gender	3.76	.063	.126

The same analysis was performed for the grey matter corrected blood flow measure (gmCBF). As revealed in Table 3, in both groups there was a significant main effect of gender, whilst age did not contribute significantly. On average, females had higher average gmCBF than males in both groups (younger females 12.4ml/100g/min higher; older females 11.1 ml/100g/min higher) when the effect of age was controlled for. The younger group model had an R^2^ of .39 and the older group model had an R^2^ of .42.

**Table 3:** Univariate GLM for each age-group, showing age and gender main effects on gmCBF.

Age group		F	p	Partial Eta Squared
Younger	Age	.09	.769	.003
	Gender	16.76	**.000**	.374
Older	Age	2.59	.120	.091
	Gender	12.28	**.002**	.321

Univariate GLM showed that there was a significant main effect of gender on gmCMRO_2_ in both age groups, but age did not have a significant effect. On average females had higher gmCMRO_2_ than males in the same age group when the age effect was controlled (younger = 38.7μmol/100g/min higher; older = 24.1μmol/100g/min). R^2^ for the younger model was .41, and the older model R^2^ was .22. Table 4 shows the results.

**Table 4:** Univariate GLM for age-groups separately, showing age and gender main effects on gmCMRO_2_ .

Age group		F	p	Partial Eta Squared
Younger	Age	.26	.618	.009
	Gender	18.27	**.000**	.395
Older	Age	.75	.393	.028
	Gender	7.33	**.012**	.220

Univariate GLMS performed for venous oxygenation showed that in the younger adult group there were no significant main effects of gender (p = .61) or age (p = .83) on Y_v_ (Model R^2^ = .01). The older group showed a significant main effect of age (F (1, 26) = 10.09, p = .004), but not gender (p = .68) on Y_v_, R^2^ = .31. In older adults, parameter estimates showed that for each additional year of life Y_v_ decreased by .46% when gender effects were controlled.

### Relationships between physiological measures and cognitive performance

Several univariate GLMs were performed to assess the contribution of physiological processes to memory and attention in each age group separately. Each model assessed the main effects of age, gender and years of education, and the physiological parameter in question, on the cognitive composite score for each age group. Ratios of tCBF: Y_v_ and gmCBF: Y_v_ were calculated to assess the rate of change in these parameters relative to one another. The use of this measure enabled investigation of how the balance of CBF and Y_v_ changes with age. One-way ANOVA indicated that there were no significant differences in the ratio of tCBF: Y_v_ between groups (F (1, 59) = 11.52, p = .097) or in the ratio of gmCBF: Y_v_ between groups (F (1, 59) = .075, p = .79) indicating that the two parameters decrease at roughly the same rate in each age group, i.e. as tCBF/ gmCBF decreases, there is a concomitant decrease in Y_v_.

#### Memory, blood flow and ratios of blood flow to venous oxygenation

There were no significant effects of tCBF, age group, gender or years of education on memory score for either younger or older adults. S1 Table in the Supplementary Material shows the F, p and partial eta squared values for separate univariate GLMs for tCBF. Likewise, there were no significant effects of gmCBF, age, gender or years of education on memory score in either younger or older adults. S2 Table in Supplementary Material shows the values of statistical tests for gmCBF and memory. The ratios of tCBF and gmCBF to Y_v_ were found to have no significant impact on memory scores for either age group.

#### Attention and tCBF

There were no significant effects of tCBF, age, gender or education on attention score in the younger group. In the older group however, tCBF had a highly significant main effect on attention, as did education. Parameter estimates for the older group revealed that for each additional 10ml/min of blood flow, reaction time on the attention tasks increased by approximately 2.2msecs on average when the demographic characteristics were controlled. R^2^ for the younger group was .08, whilst the older group R^2^ was .46. Table 5 below shows the results.

**Table 5:** Univariate GLM showing age, gender, education and tCBF main effects on attention for younger and older adults.

Age group		F	p	Partial Eta Squared
Younger	Age	1.35	.257	.055
	Gender	.28	.605	.012
	Education (years)	.05	.833	.002
	tCBF	.85	.365	.036
Older	Age	3.37	.081	.138
	Gender	1.22	.282	.055
	Education (years)	7.87	**.011**	.273
	tCBF	8.56	**.008**	.289

#### Attention and gmCBF

There were significant main effects of gmCBF and years of education on attention score in the older group, yet none of the variables contributed to attention score significantly in the younger group. Parameter estimates for the older group revealed that for each additional 10ml/100g grey matter/min of blood flow, reaction time on the attention tasks increased by approximately 34.8msecs on average when the demographic characteristics were controlled. R^2^ values were .07 for the younger group, and .43 for the older group. Table 6 displays the results.

**Table 6:** Univariate GLM showing age, gender, education and gmCBF main effects on attention for younger and older adults.

Age group		F	p	Partial Eta Squared
Younger	Age	1.20	.285	.049
	Gender	.36	.554	.015
	Education (years)	.04	.852	.002
	gmCBF	.51	.482	.022
Older	Age	2.36	.139	.101
	Gender	2.09	.163	.090
	Education (years)	8.33	**.009**	.284
	gmCBF	6.97	**.015**	.249

#### Attention and tCBF: Y_v_ ratio

Univariate GLM revealed that in the older group the ratio of tCBF: Y_v_ significantly contributed to attention score, as did years of education, see Table 6 for statistics. Parameter estimates for the older group revealed that for each additional unit of tCBF: Y_v_, attention score increased by approximately 15msecs on average when age, gender and education were controlled. Age and gender did not predict any significant amount of variance. None of the variables contributed to attention score significantly in the younger group. R^2^ values were .06 for the younger group, and .45 for the older group. Table 7 displays the results.

**Table 7:** Univariate GLM showing age, gender, education and tCBF: Y_v_ main effects on attention for younger and older adults.

Age group		F	p	Partial Eta Squared
Younger	Age	1.14	.298	.047
	Gender	.21	.653	.009
	Education (years)	.01	.911	.001
	tCBF: Y_v_	.37	.551	.016
Older	Age	1.48	.237	.066
	Gender	1.04	.321	.047
	Education (years)	8.10	**.010**	.278
	tCBF: Y_v_	7.76	**.011**	.270

#### Attention and gmCBF: Y_v_ ratio

Univariate GLM revealed that in the older group the ratio of gmCBF: Y_v_ significantly contributed to attention score, as did years of education. Age and gender did not predict any significant amount of variance. Parameter estimates for the older group revealed that for each additional unit of gmCBF: Yv, attention score increased by approximately 217msecs on average when age, gender and education were controlled. None of the variables contributed to attention score significantly in the younger group. R^2^ values were .05 for the younger group, and .40 for the older group. Table 8 displays the results.

**Table 8:** Univariate GLM showing age, gender, education and gmCBF: Y_v_ main effects on attention for younger and older adults.

Age group		F	p	Partial Eta Squared
Younger	Age	.99	.329	.003
	Gender	.07	.795	.041
	Education (years)	.00	.965	.000
	gmCBF: Y_v_	.05	.834	.002
Older	Age	1.70	.428	.030
	Gender	.65	.206	.075
	Education (years)	8.42	**.009**	.286
	gmCBF: Y_v_	5.72	**.026**	.214

#### Cognition, gmCMRO_2_ and Y_v_

Neither memory nor attention scores were significantly related to gmCMRO_2_, age, gender or education in either the younger or the older group. It was also found that there were no significant effects of Y_v_, age, gender or years of education on either memory or attention score.

### Summary

This study revealed the main findings that cognitive performance was significantly reduced in older compared to younger adults; Older adults had significantly lower tCBF and grey matter volume compared to younger adults, yet there were no differences between the groups on measures of gmCBF and gmCMRO_2_, whilst the difference in Y_v_ was approaching significance; In the older group, tCBF was marginally associated with age, and this association was attenuated when corrected for grey matter volume; In the older group tCBF, gmCBF, tCBF: Y_v_ ratio and gmCBF: Y_v_ ratio were associated with reaction times on attention tasks when age, gender and education effects were controlled, yet no such associations were observed in the younger group; In the older group, tCBF and the ratio of tCBF: Yv contributed greater amounts of variance to attention score than gmCBF in adjusted models; There were no significant contributions to memory tasks in either group, with any parameter.

## Discussion

The present study investigated the relationships between brain oxygen metabolism, blood supply and cognitive function between healthy younger and older adults. It was hypothesized that age-related declines in CBF, CMRO_2_ and cognition would be evident. Total blood flow in the brain decreased with age; however when this was corrected for grey matter volume, grey matter blood flow did not differ between young and older adults. The rate of cerebral oxygen utilization did not differ between groups, and the amount of oxygen extracted from the blood showed a trend toward being increased in the older group compared to younger. Taken together, these results suggest that there is some imbalance between the supply, consumption and demand for oxygen in the brain with older age, a pattern demonstrated in previous research [50]. A further aim of this study was to investigate the association of both CBF and grey matter oxygen utilization with performance on specific cognitive domains of attention and memory in healthy younger and older adults.

Advanced age in this sample was characterized by poorer performance on both the attention and memory measures of cognition and a reduced supply of blood to the neural tissue, yet when corrected for grey matter volume this reduction in CBF was no longer apparent. This was coupled with a trend toward increased oxygen extraction in the older group. In the older group total CBF, grey matter CBF and the ratios of both total and grey matter CBF to venous oxygenation contributed to attention score. Higher blood flow and ratios were associated with slower reaction times. Memory function was not associated with any physiological variable in the either group, and no relationships between physiological measures and cognitive performance were observed in the younger group.

### Age differences

#### Whole brain CBF was reduced in the older group, yet grey matter CBF was comparable between groups

As hypothesized, total CBF was reduced in older adults compared to younger. These findings are supported by numerous reports of MRI-based [33–35, 44, 49, 51, 52] and specifically phase contrast MRI research [48, 53]. However, when total flow was normalized to the volume of grey matter, blood flow did not differ between groups. It is understood that cerebral grey matter undergoes significant atrophy with age [54], a finding corroborated in the present data. To account for this atrophy, we corrected for tissue volume in our estimation of gmCBF. Once this volume reduction was accounted for, blood flow in the remaining grey matter was found to be unchanged over the lifespan. This result, despite being in line with some previous findings (e.g.[21, 50]), was unexpected, as grey matter CBF is generally understood to decline with age, though CBF reduction across the brain is heterogeneous across brain regions [35]. Thus, the current data may have failed to observe this decline by assessing gmCBF as a derivative of grey matter volume, rather than regionally. According to the data presented here, we can infer that while the total flow entering the brain may decrease with age, blood supply to grey matter specifically does not change *per se*. This finding, coupled with age-related decline in grey matter volume, suggests that the observed decrease in total flow may be due to an overall reduction in volume with aging.

It was observed that there were differing patterns of age-related changes between the groups. In the younger adults, there were no observed alterations of any vascular measure with age, yet the older group showed significant age-related decreases in both blood flow and venous oxygenation, whilst gmCMRO_2_ remained the same. This pattern of hemodynamic changes suggests that in older age, neural tissue extracts greater amounts of oxygen from the blood when the supply is reduced, in order to maintain the same level of oxygen consumption.

#### CMRO_2_ did not differ between age groups

Cerebral metabolic rate of oxygen use did not differ between age groups. Previous PET studies of brain aging and CMRO_2_ have been inconsistent. Most often oxygen utilization is observed to decrease with age [16, 20–28], yet some studies have found an increase in oxygen demand with age [32, 33], and, in-keeping with the current findings, other researchers have reported no change in CMRO_2_ over the lifespan [21, 29, 30, 40, 55, 56].

The majority of this previous research utilized PET technology, making correction for brain parenchyma volume reduction difficult and possibly resulting in underestimation of CMRO_2_. Developments in MRI capabilities allow for greater sensitivity in detecting partial volumes and accounting for brain atrophy, thus providing more robust estimation of oxygen use [57]. The method employed in this study calculated the rate of oxygen use relative to the size of the grey matter on an individual basis to overcome this potential limitation.

Lu et al. [33] reported that CMRO_2_ increased across the lifespan; however potential age-related changes in hemoglobin concentration were not accounted for. The ability for blood to carry oxygen, and thus the hemoglobin content, can decrease by a rate of 0.0079mmol/L per year [21]. Researchers [32] re-analysed the data from Lu et al. [33] to include the potential age-effect on hematocrit to demonstrate that the CMRO_2_ dependence on age was no longer significant. Therefore, the present study adjusted for the effect of age on hemoglobin content, replicating this result, which suggests that some previous findings may have overestimated the change in CMRO_2_ that is truly attributable to aging rather than that which is dependent on age-associated hemoglobin decrease.

#### Hemodynamic differences between groups

Consistent with others [33, 50, 58], oxygenation of the venous blood declined with age in older adults (i.e. an increase in the oxygen extraction fraction (OEF)), and this was coupled with a decrease in cerebral blood flow, while CMRO_2_ remained constant. Overall decrease in blood supply without a proportionate fall in metabolism would likely have this result [50]. The ratio of tCBF: Y_v_ was trending towards a decline in the older group as compared to the younger adults, suggesting that the equilibrium between oxygen demand and whole brain blood supply is gradually altered across the lifespan. This is possibly due to diminished efficiency of the neural tissue with aging; the brain tissue requires more oxygen from the blood supply to maintain the availability of oxygen in the brain parenchyma, and thus, energy homeostasis. Previous research by Dastur et al. [50] using the nitrous oxide method [59] showed a similar pattern of results wherein CBF and CMRO_2_ remained the same with age, matched with a trend toward decreased venous oxygenation. The authors concluded that oxygen extraction from the arterial blood is increased over the lifespan in order to maintain optimum CMRO_2_ levels due to relative circulatory insufficiency. Note however, that no correction for brain volume was performed in this previous work, limiting the comparability of results with the present findings, in which total CBF was reduced in the older group compared to the younger, but CBF specific to grey matter tissue did not differ between groups. Moreover, the ratio of grey matter blood flow to venous oxygenation was not statistically different between the two age groups; these two parameters thus vary at the same rate with age. This highly non-significant result would indicate that the loss of gmCBF is in line with oxygen extraction in both younger and older adulthood. This suggests that in normal, healthy aging the cerebrovasculature is functioning beyond what is required to compensate for decreases in flow and oxygen extraction, particularly when adjusted for the grey matter atrophy.

Regardless, if the ability of the circulatory system to adjust to local changes in supply and requirement for oxygen is compromised by age-related pathologies such as hardening of vessels or accumulation of arteriosclerotic plaques, then increased extraction of oxygen from the circulating blood is the last remaining mechanism that can accommodate further demand, even when blood flow is maintained.

#### Summary of age group differences

The current data showed a decrease in supply of blood across the whole brain, yet flow and oxygen metabolism in the grey matter alone were maintained. This was coupled with a trend toward an increase in oxygen extraction between the age groups. This gradual shift in maintenance of energy homeostasis was accompanied by declining grey matter volume. The measured decrease in total CBF appears to be primarily due to atrophy of cortical tissue, rather than a true decline in flow. Overall, the data shows that aged brains have less volume, yet receive the same amount of blood flow per 100g of tissue as younger. This, matched with the observed trend towards an increase in oxygen extraction from the blood, suggests that the aged brain requires greater amount of oxygen per unit of tissue in order to maintain optimum metabolism.

### Association between vascular factors and cognitive performance

The present study expanded on previous research by assessing the contributions of vascular and metabolic factors to attention and memory in physically and cognitively healthy adults. Consistent with previous research, attention and memory performance were lower in older adults compared to younger [3, 5]. Statistical analyses showed differences in the contributions of vascular factors to attention performance between the younger and older groups. While in the older group there were significant associations of CBF and the ratio of CBF to venous oxygenation (a measure of the coupling of flow and consumption) to the attention composite measure, these relationships were not observed in the younger adults. Memory was not associated with any physiological parameter in either group. This is in support of findings using phase contrast MRI to show a task-specific CBF- cognition relationship, wherein CBF predicted attention and information processing speed [48, 52], but not memory measures [60].

In the older group, when adjusted for participant characteristics, attention task performance was inversely related to blood flow, such that faster responses were associated with lower blood flow. While these findings are consistent with previous research in healthy participants [e.g. 48, 52], this is opposite to the expected outcome, given that both CBF and cognitive performance decrease with age.

This finding is in line with those of previous healthy aging studies. For example, it was found that blood flow in the carotid and basilar arteries in healthy older adults was negatively correlated to cognitive processing speed [48]. Bertsch et al. [52] reported a similar inverse relationship between resting grey matter blood flow and selective attention and tonic alertness in healthy individuals. The authors related their findings to the hypothesis of neural efficiency [61]. This theory posits that general intelligence is not a reflection of how hard a brain works, but due more to proficiency of neural circuits, as less ‘fuel’ i.e. oxygen and nutrients from the circulation, is required when the brain is working more efficiently. Faster reaction time on the attention tasks is assumed to represent more efficient processing. Attention and intelligence measures have been shown to be highly associated [61, 62], lending support to the notion that better performance on attention tasks could be linked to lower global blood flow.

Another study reporting significant correlations between cognition and resting CBF found that once brain size was taken into account these associations disappeared [63]. In the present study, global CBF predicted attention score, yet this association was attenuated when volume of grey matter was accounted for, suggesting that some of blood flow effects are partially due to brain atrophy [47]. Likewise, in the present research the ratio of total CBF: venous oxygenation was found to predict attention score (a higher ratio was associated with slower responses on attention tasks) when age, gender and education were controlled, however this relationship was statistically weaker when corrected for grey matter volume. While this is difficult to interpret, a possible explanation is that oxygen extraction may be driving this association; a lower venous oxygen percentage (i.e. higher oxygen extraction) for the amount of blood flow could signify that the brain tissue is more at risk, and this would present as a smaller ratio. The attenuation of the relationship between CBF: Y_v_ ratio and attention score once brain tissue volume is accounted for suggests that at least some portion of this flow/consumption coupling impact is due to grey matter volume differences.

#### CMRO_2_ and Y_v_ did not predict cognition

Contrary to expectations, grey matter CMRO_2_ did not contribute to either memory or attention score, which could be the result of the compensation between CBF and Y_v_. These findings have been corroborated in previous literature in studies of healthy aging [40, 63–65]. One study [64] found no association between cerebral metabolism of glucose and visual memory performance in healthy adults. Researchers concluded that resting brain metabolism is not linked with mental ability in the absence of disease. This notion is corroborated by Dastur et al. [50], who found a connection between mental abilities and CMRO_2_ in normal older men with asymptomatic, sub-clinical vascular disease, but not in men who were optimally healthy. The present study employed a rigorous health screening questionnaire to exclude those presently or previously suffering disease or poor health, lending support to these hypotheses. Similarly, oxygenation of the venous blood was not associated with either cognitive measure. Previous research has linked lower oxygen content in the jugular vein to post-operative cognitive dysfunction in patients [66], however this is the first study to examine the association of venous oxygenation and cognitive performance in a healthy population of younger and older adults.

#### Summary of vascular and metabolic contributions to cognitive performance

Taken together, these results suggest that declining blood supply and grey matter volume results in increased extraction of oxygen from the blood in order to maintain normal oxygen consumption, a process that likely protects against neural impairment. Cognitive performance slows even in healthy aging, and there are differences in the contribution of vascular factors to cognition from younger to older adulthood. Physiological factors were not found to contribute to cognitive performance in the younger group. In older adults however, oxygen supply has an inverse effect on attention when demographic characteristics are accounted for, such that lower flow is associated with faster cognitive speed, perhaps indicating better neural efficiency. Furthermore, the ratio of blood flow to oxygen extraction also contributes to attention score. Interestingly, these relationships are attenuated when grey matter atrophy is corrected for, indicating that for these older, cognitively intact individuals, brain volume has some impact on cognitive performance.

### Limitations

The findings of the current study should be interpreted in light of some limitations. Participants were both healthier and more educated than what may be expected in the general population of adults, and were selected for the purposes of reducing any unwanted confounding variables in terms of biological aging. Care was taken to exclude individuals with cardiovascular risk factors that may confuse findings resulting from normal healthy aging.

Much of the previous research on CBF with aging has employed analysis based on brain region, due to the acknowledged heterogeneity of blood flow across different brain areas [21]. Previous studies indicate that there is a greater decrease in CMRO_2_ in the grey compared to white matter across the lifespan [67], and others [68] reported that CBF and CMRO_2_ reductions occur primarily in the parietal and temporal cortices, with other areas remaining unaffected. The method employed in the present study is considered to be valid and reliable for whole-brain estimation of CBF [63], and CMRO_2_ [32], yet does not allow for regional analysis to be performed. Measurement of grey matter CMRO_2_ without analyzing by specific region could underestimate the alteration in grey matter oxygen use whilst overestimating the oxygen use in the white matter, thus cancelling out any age effect in some regions versus others. The current study corrected for this by using the VBM toolbox in SPM12 (SPM12, University College of London, UK) which uses an iterative method to optimally segment different tissue types of the brain to calculate CMRO_2_ in the grey matter only. However, this approach doesn’t allow for the distinction between grey and white matter contributions to the aging effect in vascular and metabolic function. If there is a differential rate of change in tissue volume with age, and this leads to changes in the ratio of grey to white matter across the lifespan, then CMRO_2_ could appear to be modified without any true change in either grey or white matter rate of oxygen use, yet even after accounting for this gmCMRO_2_ was not found to change across the lifespan, a finding corroborated in the literature [24]. The data showed that both grey and white matter was reduced in the older group compared to the younger adults, yet only the grey matter difference was significant. It has been suggested that alterations of tissue microstructure are the more commonly detected age-related change in white matter over volume reduction, and these structural changes are not easily observed using conventional T1-weighted imaging [69].

Additionally, the CMRO_2_ values obtained in this study show some discrepancy when compared to literature values (e.g. 146 μmolO_2_/100g/min on average in the younger group in the current work, versus 171μmolO_2_/100g/min for an average 20 year old male reported in Aanerud et al. [21], versus ~135μmolO_2_/100g/min for healthy controls reported in Thomas et al [70]). Design differences between these studies (e.g. samples, measurement tools, variation in haematocrit values) would limit the extent to which the specific values of CMRO_2_ can be compared.

CBF change over the lifespan is heterogeneous across different brain regions, and distinct areas of the brain may be more responsible for cognitive performance than others [60, 63]. Cerebral hypoperfusion can result in neural deterioration, and subsequently impaired cognition; however it is also possible that brain atrophy causes hypoperfusion due to decreased demand for oxygen and nutrients. The cross-sectional design of the study limits the extent to which causality can be deduced, further study is required to address whether blood flow changes are a cause or a consequence of brain shrinkage with age.

### Conclusion

In summary, the current research showed that healthy aging is linked to inferior memory and attention, reduced total cerebral blood flow and a trend towards increased oxygen extraction, whilst blood flow and oxygen metabolism in the grey matter is maintained. These results indicate that there is some imbalance between the demand and the supply for oxygen and nutrients in the brain, even in successful aging. CBF was shown to contribute to attention task performance, though this relationship was in an unexpected direction with higher flow associated with poorer performance. The results of this study suggest that the association between CBF and cognition is age-dependent and task-specific. Overall deterioration in the aging brain results in losses of synaptic and vascular density and viability, and these diminishing functions may be responsible for the progressive slowing of cognitive speed, particularly in the realm of attention. Understanding how vascular and metabolic factors impact on cognition in normal, healthy adults is essential to develop efficient therapies to prevent and treat cognitive losses in older age. Future research employing regional brain area analyses and incorporating larger sample sizes is necessary to continue to investigate these relationships, in addition to the inclusion of cognitively impaired elderly to assess whether there are differential associations between CMRO_2,_ Y_v_, CBF and cognitive performance between impaired and healthy aging.

## Materials and methods

### Participant characteristics

Vascular, metabolic and cognitive function was assessed in 60 healthy (active, asymptomatic, independently-living) adults. Two separate age groups were formed, the younger group <50 years (n = 31, ages 21–44, M 29.97 years, SD 6.17, 13 females) and the older aged >50 (n = 29 ages 55–75, M 65.00 years, SD 5.56, 16 females). Exclusion criteria included contraindications for MRI, left handedness, existing or previous cardiovascular or psychiatric disorders, blood pressure above 140/90mmHg, diabetes, pregnancy, use of medication for cardiovascular, neurologic or psychiatric disorders, and any other chronic illness. A telephone-based assessment (the Modified Telephone Interview for Cognitive Status- TICS-m [71]) was used to screen for mild cognitive impairment and dementia for participants aged over 45 years. Participants scoring 23 or below were omitted from the final analysis (n = 3). After receiving an explanation of the study procedure, participants provided written informed consent. This study was granted ethical approval by: Alfred Hospital Ethics Committee (338/13) and Swinburne University Human Research Ethics Committee (2013/316). Participant characteristic information is presented in Table 1.

### Experimental design and procedure

Data was acquired in one testing session, approximately 3 hours length. Each participant had their blood pressure taken by a registered nurse, and then underwent a magnetic resonance imaging (MRI) scan. A 30-minute computerized cognitive assessment followed.

#### MRI data acquisition

Data was collected using a 3T Siemens Tim Trio MRI system (Siemens, Erlangen, Germany) fitted with a 32-channel head coil at Swinburne University, Hawthorn, Australia. Participants were requested to minimize head movements, and foam padding was inserted around the head to aid this.

#### Total and grey matter cerebral blood flow

Phase contrast MRI uses the phase of acquired images to determine the velocity of moving spins to obtain quantitative whole-brain blood flow measurements. Based on the maximum intensity projection reconstruction of the TOF angiogram, each of the four feeding arteries of the brain (left and right internal carotid arteries and left and right vertebral arteries) were scanned separately [72]. Scans were oriented perpendicular to the arteries, and positioned with the center of the FOV placed at the center of each target vessel Imaging parameters were: single slice, peripherally gated, TR 41.3ms, TE 4ms, FA 25°, FOV 160 x 160 x 5mm^3^, voxel size 0.5 x 0.5 x 5mm^3^, maximum velocity encoding 100cm/s. The flow (velocity x area) through the four arteries was summed together and multiplied by 60s/min to give the total CBF (tCBF) in ml/min. Mean grey matter CBF (gmCBF) was calculated by dividing the total CBF by grey matter volume relative to white matter volume. White matter flow was assumed to be 40% of grey matter flow [25]. This ratio and the grey and white matter volumes obtained from the segmentation procedure were used to calculate tissue-specific flow and metabolism rates. Equations for gmCBF are supplied in Supplementary Material.

#### Measurement of gmCMRO_2_

Grey matter CMRO_2_ was estimated due to differing rates of volume loss and oxygen metabolism between tissue types with age. This study employed a technique developed by Xu et al. [73], improved upon by Liu et al. [72], using independent MRI estimates of venous blood oxygenation (Y_v_) and cerebral blood flow. A 30-second baseline of arterial oxygen saturation was acquired with a pulse oximeter and averaged for each participant to give the value of Y_a._ Total CBF (tCBF) was measured using phase-contrast MRI and Y_v_ was estimated using T2- relaxation-under-spin-tagging (TRUST) [74]. Grey matter CBF (gmCBF) was calculated from tCBF values as shown in Supplementary Material. Fick’s principle of arteriovenous difference [75] was used to quantify the rate of cerebral oxygen metabolism as per previous research [33, 76–79]. The equation was adapted for grey matter:

gmCMRO2=gmCBF.(Ya−Yv).Ca [1]

Where gmCBF is the cerebral blood flow in ml/100g grey matter/min, Y_a_ and Y_v_ are the percentage of oxygen saturation of the arterial and the venous blood respectively, and C_a_ is the blood’s oxygen carrying capacity, generally established in the literature as 833.7 μmol of O_2_/ 100ml of blood for a hematocrit level of 0.44 [80]. Previous research [21] reports age- and gender-dependence in hemoglobin concentration. To overcome this confound, C_a_ was set at856 μmol of O_2_/ 100ml blood for young males and 815 μmol of O_2_/ 100ml of blood for young females, for hematocrit levels of 0.42 and 0.40 respectively, as per previous research [80], and was adjusted for the decline rate of 0.0079 μmol of O_2_/ml/year [21]. This value had little effect on the calculation as a whole; experimental substitution with a single average value of 833.7 μmol of O_2_/ 100ml of blood for all participants did not affect the outcome of statistical analyses.

All data processing was performed off-line with MATLAB 2014b (The MathWorks Inc., Natick, MA, 2014) using SPM12 (SPM12, University College of London, UK) or software developed in house. MATLAB scripts were used to segment the T1-weighted images of the brain into grey and white matter volumes using SPM12.

A high-resolution T1-weighted image was acquired using a 3D magnetization-prepared rapid gradient-echo (MPRAGE) image (axial, TR 2300ms, TE 2.52ms, voxel size 1 x 1 x 1mm^3^, FOV 256mm, 176 slices) to provide an estimation of the intracranial, grey, and white volumes for calculation of blood flow per unit mass of grey matter tissue. This accounts for variance in brain size and atrophy across participants.

**Fig 1:**
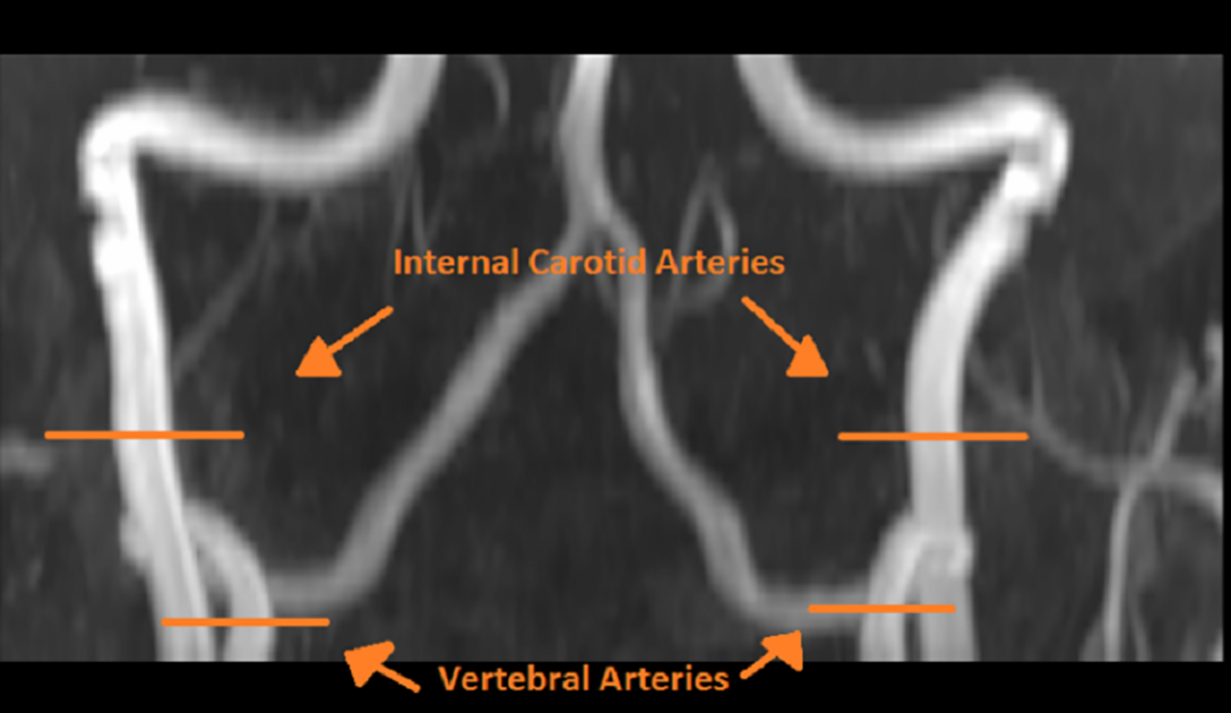
Location of phase contrast scans for the left and right ICA and VA.

A whole-brain 3D time-of-flight (TOF) angiogram enabled visualization of the four feeding arteries of the brain for phase-contrast MRI slice positioning (Fig 1). Imaging parameters were TR 20ms, TE 3.59ms, flip angle 18°, FOV 200 x 180 x 1mm^3^, voxel size 0.5 x 0.5 x 1mm^3^, 149 slices.

#### Global venous oxygenation

**Fig 2:**
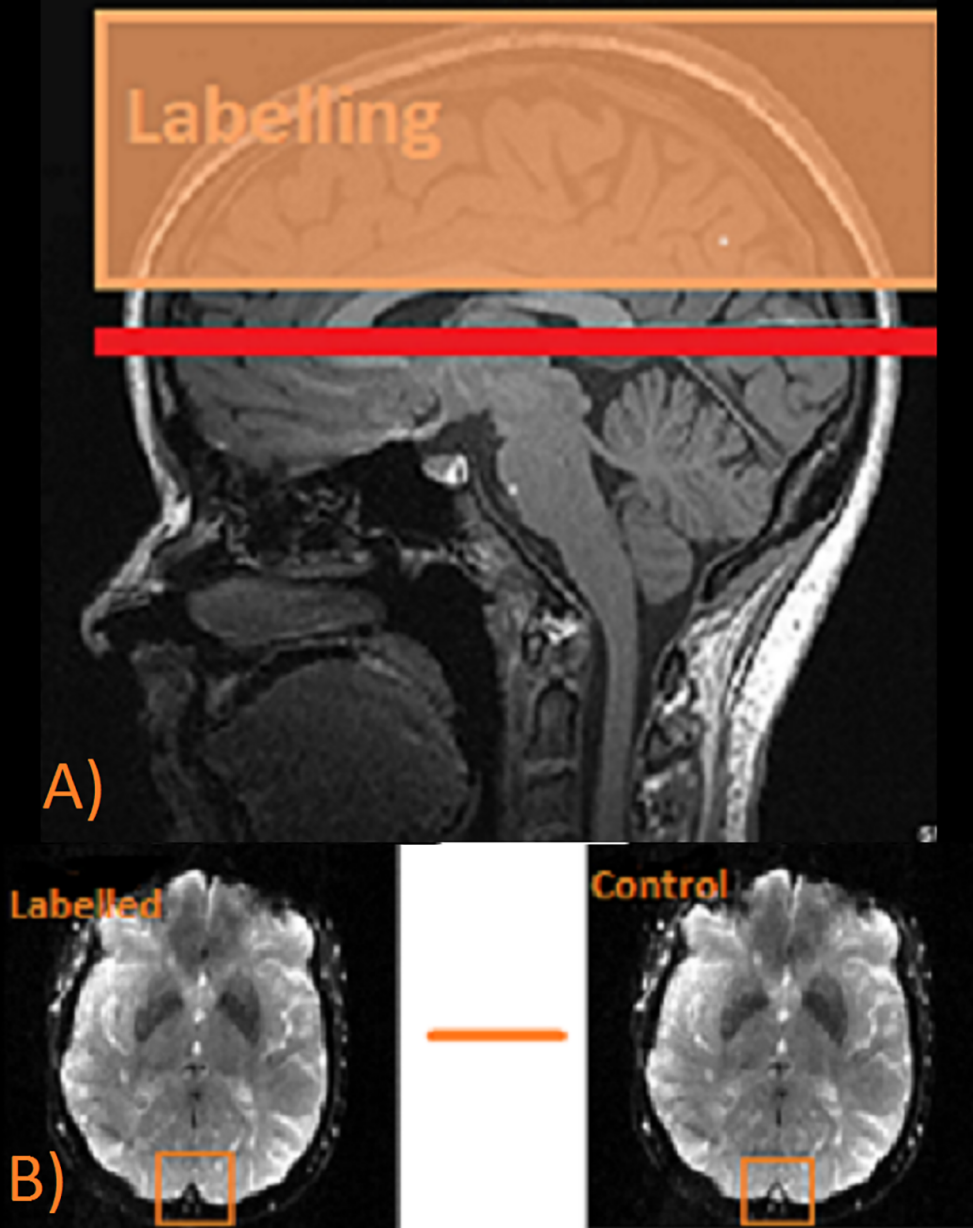
Quantification of the venous oxygenation using T2- relaxation-under- spin- tagging (TRUST) MRI **A)** The location of the labeling slab region (orange block) of the TRUST scan upstream from the imaging slice (red line). T2 blood signal was acquired from the superior sagittal sinus (SSS) **B)** Raw axial images of the control and labelled scans, SSS region is shown in the orange box. The label inverts the venous blood signal so it appears dark in the image.

TRUST quantitatively estimates the oxygenation of the venous blood by measurement of pure blood T2 in the superior sagittal sinus (SSS). Imaging parameters: single-shot echo-planar imaging (EPI), axial plane, TR 4000ms, TI 1400ms, four effective TEs 0, 40, 80, 160ms; tagging thickness 50mm, gap 25mm, voxel size 1.4 x1.4 x 5mm^3^, 1 average, scan duration 2.20 min. The slice was parallel to the anterior- commissure posterior-commissure (AC-PC) line with a distance of approximately 1cm above the confluence of sinuses. Slice positioning was based on a middle sagittal survey image (Fig 2A). The control and tagged images were subtracted pairwise to yield the venous blood signal, which was fitted a mono-exponential function to obtain the *T*_*2*._ The *T*_2_ was then in turn converted to Y_v_ via a calibration plot as described previously [74, 81, 82].

#### Computerized cognitive assessment

Cognitive function was measured using the Swinburne University Computerized Cognitive Assessment Battery (SUCCAB) [83]. This validated computerized test battery of 8 tasks is designed to capture the range of cognitive functions that decline with age. Tests were administered in the following order: simple reaction time, two-choice reaction time, immediate recognition memory (testing immediate memory of abstract images), congruent and incongruent color-word Stroop tasks, spatial working memory (testing working memory of spatial locations), contextual memory (testing recall memory of objects in spatial locations), and delayed recognition memory (testing the delayed recall of images from the immediate recognition memory component). Details of the tasks have been described previously [83]. Participants completed the SUCCAB once with duration of approximately 30 minutes. Participants responded to each task using a button box [83]. The researcher read the instructions aloud to ensure consistent explanation of all tasks. Each task was preceded by a brief practice trial. Memory and attention composite scores were calculated from the SUCCAB. Response times for immediate and delayed recognition, spatial working and contextual memory tasks were averaged to give the memory composite score. Simple and choice reaction time, and the two Stroop task response times were averaged to give the attention composite score. The variables are weighted such that greater values indicate slower processing times. The method of composition has been described previously [84].

#### Modified Telephone Interview for Cognitive Status (TICS-m)

The TICS-m is a reliable and validated brief 13-item test of cognitive functioning that is administered over the telephone [71]. The items covered related to questions of orientation, repetition, naming and calculations, with scores ranging from 0 to 35. The modified version of the TICS also includes immediate and delayed recall. Participants who scored 23 or below were omitted from the final analysis (n = 3).

#### MRI data analysis

An in-house Matlab script was used for quantification of CBF, which involved a region-of–interest (ROI) mask being manually drawn onto each target artery based on the magnitude image of each PC MRI scan. The phase signals (i.e. velocity values) within the masks were summed and combined with the cross-sectional area of each artery to calculate blood flow in ml/min for each artery. Blood flow values of each artery along with the diameter to convert to blood flow (ml/min). Velocities of the four arteries were added together to give the total CBF. Brain volume differences were accounted for by normalizing total CBF in ml/min to total grey matter (Vgm) and white matter (Vwm) in grams to give estimates of CBF in the grey and white matter separately. Grey and white matter volumes were estimated from the T1 structural MPRAGE scan using voxel-based morphometry (VBM8) segmentation with SPM12 software (SPM12, University College of London, UK). CMRO_2_ per unit mass of grey matter was calculated according to Eq 1 above. Eq 1 was used to obtain unit-mass grey matter CMRO_2_ μmol of O_2_ per 100g of grey matter per minute as per previous literature [32].

### Statistical analyses

IBM SPSS statistics v.23 (Chicago, IL, USA) was used to conduct analysis. Means and standard deviations for patient characteristics, baseline vascular and metabolic (tCBF, gmCBF, Y_v_ and gmCMRO_2_) structural markers (ICV, Vgm and Vwm), and cognitive composite scores are shown in Table 1 for the whole sample and separately by age group. Differences between age groups were assessed using one-way independent samples analyses of variances (ANOVAs). Univariate general linear models (GLMs) were used to assess the main effects of age and gender on physiologic variables for each age group separately.

Separate univariate GLMs were performed for each age group to assess the main effects of vascular and metabolic factors to the cognition measures separately. Age, years of education and the vascular, ratio or metabolic factors were entered as fixed factors, and age was entered as a covariate. P values of 0.05 or less were considered statistically significant in all analyses; however a correction for multiple comparisons (Bonferroni’s correction) was applied to adjust for the inclusion of two physiological measures in the hypotheses. Significance level was adjusted for two comparisons in the univariate linear regression analyses (p≤ 0.025). P values between 0.06 and 0.05 were considered to be marginally significant prior to correction, and reported as such.

#### Calculation of grey matter cerebral blood flow (gmCBF)

Calculation of CBF in the grey and white matter on the assumption that white matter flow is approximately 40% that of grey matter as per previous literature [25]. The following equations were used to estimate gmCBF from tCBF values:

tCBFv=tCBF/(Vgm+Vwm).p [2]

tCBFv=gmCBFv+wmCBFv [3]

wmCBFv=0.4.gmCBFv [4]

tCBFv=gmCBFv+0.4.gmCBFv=1.4.gmCBFv [5]

gmCBFv=tCBFv/1.4=tCBF/(1.4.(Vgm+Vwm).p) [6]

Where Vgm and Vwm are the volumes of grey and white matter respectively in ml. ρ is the mass density of tissue used as the scaling factor to convert tissue volumes to mass, assumed as 1.06g/ml [85]. tCBFv is the volume-corrected total CBF, normalized to the volumes of grey and white matter, in ml/100g brain tissue/ min. gmCBFv and wmCBFv are the volume corrected blood flows for grey and white matter respectively. A 30-second baseline of arterial oxygen saturation was acquired with a pulse oximeter and averaged for each participant to give the value of Y_a._ tCBF was measured using phase-contrast MRI and Y_v_ was estimated using T2- relaxation-under-spin-tagging (TRUST) [74].

## Supporting information

S1 Table Table showing the results of univariate GLM on age, gender, education and tCBF contributions to memory score for younger and older adults. R^2^ values for younger adults was .17, and older adults was .10.
(DOCX)

S2 Table Table showing the results of univariate GLM on age, gender, education and gmCBF contributions to memory score for younger and older adults. R^2^ values for younger adults was .17, and older adults was .06.
(DOCX)
