# Supplementary material for: An investigation of cerebral oxygen utilization, blood flow and cognition in healthy aging
Source: PLoS One. 2018 May 22;13(5):e0197055. doi: 10.1371/journal.pone.0197055 (PMC5963791; doi:10.1371/journal.pone.0197055)
Supplement: S1 Table — R2 values for younger adults was .17, and older adults was .10. (DOCX) [file pone.0197055.s001.docx]

**Table S1**

| Age group |  | F | p | Partial Eta Squared |
| --- | --- | --- | --- | --- |
| Younger | Age | 1.07 | .312 | .041 |
|  | Gender | 1.64 | .212 | .062 |
|  | Education (years) | .02 | .894 | .001 |
|  | tCBF | .33 | .572 | .013 |
| Older | Age | .41 | .531 | .019 |
|  | Gender | .64 | .434 | .029 |
|  | Education (years) | .26 | .615 | .012 |
|  | tCBF | 2.11 | .162 | .091 |
