# Supplementary material for: An investigation of cerebral oxygen utilization, blood flow and cognition in healthy aging
Source: PLoS One. 2018 May 22;13(5):e0197055. doi: 10.1371/journal.pone.0197055 (PMC5963791; doi:10.1371/journal.pone.0197055)
Supplement: S2 Table — R2 values for younger adults was .17, and older adults was .06. (DOCX) [file pone.0197055.s002.docx]

**Table S2**

| Age group |  | F | p | Partial Eta Squared |
| --- | --- | --- | --- | --- |
| Younger | Age | .99 | .329 | .038 |
|  | Gender | .62 | .437 | .024 |
|  | Education (years) | .03 | .876 | .001 |
|  | gmCBF | .53 | .472 | .021 |
| Older | Age | .17 | .683 | .008 |
|  | Gender | .60 | .449 | .028 |
|  | Education (years) | .18 | .679 | .008 |
|  | gmCBF | 1.11 | .304 | .050 |
